# New Perspectives for Spinal Cord Stimulation in Parkinson’s Disease-Associated Gait Impairment: A Systematic Review

**DOI:** 10.3390/biomedicines12081824

**Published:** 2024-08-12

**Authors:** Christian G. Seufert, Matthias C. Borutta, Martin Regensburger, Yining Zhao, Thomas Kinfe

**Affiliations:** 1 Division of Functional Neurosurgery and Stereotaxy, Friedrich-Alexander University Erlangen-Nürnberg, 91054 Erlangen, Germany; christianseufert32@gmail.com (C.G.S.); yining.zhao@uk-erlangen.de (Y.Z.); 2 Department of Neurology, Friedrich-Alexander University Erlangen-Nürnberg, 91054 Erlangen, Germany; matth.bor@web.de; 3 Department of Neurology, Molecular Neurology, Division of Movement Disorders, Friedrich-Alexander University Erlangen-Nürnberg, 91054 Erlangen, Germany; martin.regensburger@uk-erlangen.de; 4 Department of Artificial Intelligence in Biomedical Engineering, Friedrich-Alexander University Erlangen-Nürnberg, 91054 Erlangen, Germany

**Keywords:** Parkinson’s Disease, spinal cord stimulation, gait dysfunction, motor symptoms, freezing of gait, deep brain stimulation, neurostimulation, neurodegenerative disorders, neuromodulation, movement disorders

## Abstract

Parkinson’s Disease is a neurodegenerative disorder manifesting itself as a hypokinetic movement impairment with postural instability and gait disturbance. In case of failure and/or limited response, deep brain stimulation has been established as an alternative and effective treatment modality. However, a subset of PD patients with gait impairment represents a therapeutic challenge. A systematic review (2000–2023) was performed using PubMed, Embase, Web of Science, Scopus, and Cochrane Library databases to determine the efficacy, stimulation waveform/parameters, spine level, and outcome measures of spinal cord stimulation using different waveforms in PD patients with and without chronic pain. Spinal cord stimulation responsiveness was assessed within the pre-defined follow-up period in three groups (short-term follow-up = 0–3 months; intermediate follow-up = 3–12 months; and long-term follow-up = more than 12 months). In addition, we briefly outline alternative neurostimulation therapies and the most recent developments in closed-loop spinal cord stimulation relevant to PD. In summary, 18 publications and 70 patients from uncontrolled observational trials were included, with low-quality evidence and conflicting findings. First and foremost, the currently available data do not support the use of spinal cord stimulation to treat PD-related gait disorders but have confirmed its usefulness for PD-associated chronic pain.

## 1. Introduction

The rising number of patients suffering from Parkinson’s Disease (PD) globally represents a considerable burden. The global prevalence of this disease increased from 2.5 million in 1990 to 6.1 million in 2016, corresponding to more than a doubling within less than 30 years, with a further increase in the number of cases expected. This cannot be explained by the aging population and demographic change alone, strengthening the assumption that other (environmental and epigenetic) factors may have an impact on PD incidence [1].

An analysis of disability-adjusted life years (DALYs) estimated a total of 3.2 million DALYs related to PD in 2016, corresponding to 0.5 years per affected individual lost due to death or disability associated with PD [1]. Moreover, with the improvement of dopaminergic therapies and continuous therapies controlling extrapyramidal motor symptoms, additional symptoms become more relevant, including pain and gait disability [2]. Pain, for example, has been observed in 68–95% of PD patients across all disease stages and was frequently associated with lower back pain and gait disorder [3]. Pain and gait disorder, in turn, lead to a decrease in physical activity and physiotherapy.

PD is a neurodegenerative disease that centers on the pathological protein aggregation (Lewy bodies) and the degeneration of neurons in the substantia nigra, leading to a dopamine deficiency [4,5]. It is defined as an incurable and progressive hypokinetic movement disorder with cardinal symptoms of rigidity, tremor, akinesia, and postural instability, leading to motor and gait disturbance (PIGD) in the progression of the disease. These motor symptoms are accompanied by a deterioration of non-motor functions, such as cognitive decline, sleep disturbance, depression, and dysautonomia, which in turn impair the quality of life in a considerable way [6]. To control these symptoms, especially motor symptoms, standard therapy currently includes drug therapy to modulate dopamine balance, such as levodopa, dopamine agonists, monoamine oxidase-B inhibitors, catecholamine-O-methyltransferase inhibitors, and apomorphine [5]. Along with drug therapy, complementary and supportive therapies include but may not be limited to physiotherapy, occupational therapy, speech/language therapy, and dietary advice. However, with increasing therapy duration, motor fluctuations (on/off periods) and dyskinesia may become drug-resistant in approximately 40% of PD patients [7].

Under such circumstances, deep brain stimulation (DBS) has effectively and safely demonstrated its potential usefulness as an adjunct therapy for motor fluctuation, dyskinesia, and the ineffectiveness of levodopa. However, a considerable proportion of PD patients develop deterioration of gait performance and postural instability associated with a less favorable DBS responsiveness and/or adaptation of pharmacotherapies, indicating the need for additional and adjunctive neurostimulation therapies. Treatment-resistant gait disorder is a major cause of disability in later stages of PD, predisposing patients to immobility and fall-related injuries [8,9,10]. In line with this, pain syndromes in PD are frequently not sufficiently responsive to an adaptation of dopaminergic therapy or to classical analgetic therapy [11]. In search of alternative neurostimulation therapies, specifically targeting gait parameters and chronic pain (back pain), spinal cord stimulation of the dorsum column (SCS), non-invasive brain stimulation such as transcranial magnetic stimulation (TMS), and vagus nerve stimulation (VNS) have been examined and are currently being investigated in ongoing clinical trials [12,13,14,15,16,17,18,19,20,21].

Our objective is to conduct a systematic review of the existing literature on the use of SCS in treating PD-associated gait impairment, with the expectation of finding a significant number of studies supporting its use. Second, we aimed to analyze clinical parameters, score assessment, implantation spine level, stimulation parameters, and adverse events related to SCS treatment, hypothesizing that certain parameters may be more effective than others. In addition, we compared the effects of SCS on PD patients with and without pain, hypothesizing that SCS may have different effects depending on the presence of pain, along with an assessment of the quality of evidence supporting the use of SCS in PD patients, expecting to identify the level of evidence. Lastly, we briefly discuss and compare invasive as well as non-invasive neuromodulation techniques and outline their potential mechanisms of action to identify gaps in current research and to provide suggestions for future investigation, with the aim of guiding future research in this field.

## 2. Materials and Methods

### 2.1. Search Design and Inclusion/Exclusion Criteria

This systematic review was conducted according to the PRISMA 2020 statement guidelines following PICO guidelines (P: patients; I: intervention/SCS; C: comparison; and O: outcome) for PD patients with gait impairment (P), who received SCS (I) with the evaluation of potential predictive factors (C) that were related to the SCS outcome (O) [15]. A structured literature search was performed using PubMed, Embase, Web of Science, Scopus, and Cochrane Library databases. Manual library research was independently performed by 3 reviewers. The following keywords were used: Parkinson’s Disease (PD), spinal cord stimulation (SCS), deep brain Stimulation (DBS), motor (symptoms), freezing of gait (FOG), gait impairment, postural instability, neurodegenerative disorders, neuromodulation, and movement disorders. Articles published from January 2000 to January 2023 were considered.

**Figure 1:**
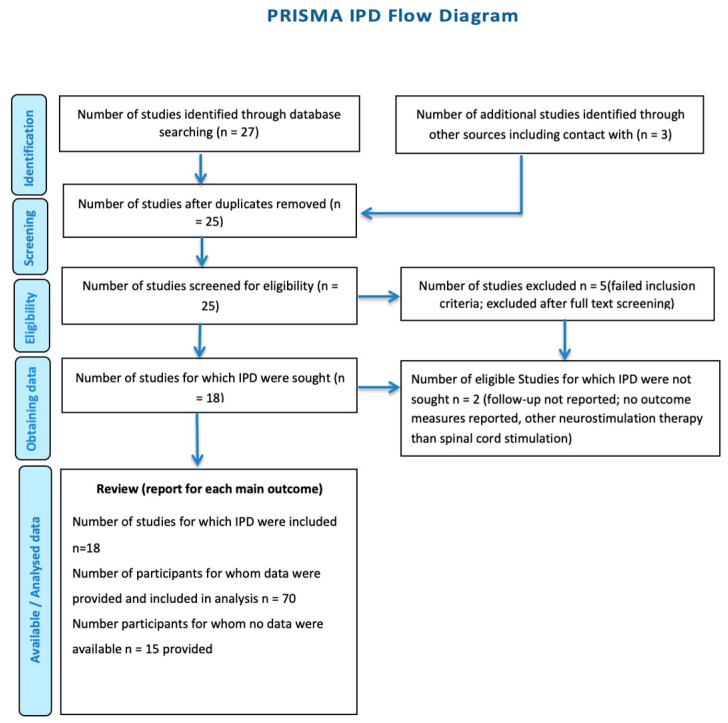
The PRISMA flow chart of the assessed in-human studies related to SCS for PD-associated gait disorders in Parkinson’s Disease.

An additional inclusion criterion was at least one follow-up visit with clinical testing of gait parameters. Individual patient data, such as age, gender, medications, the MDS United Parkinson Disease Rating Scale (UPDRS(-III)), the x-meter walk test, the Freezing of Gait Questionnaire (FOG-Q), the Timed Up and Go test (TUG), the SWS test, the Hoehn Yahr Score (H&Y), the Visual Analog Scale (VAS), the Short-Form McGill Questionnaire 2 (SF-MPQ-2) (pain), and disease duration, as well as the stimulation spine level and parameters, were extracted. The stimulation parameters were separated into the location of stimulation, frequency, intensity, and duration. The reported follow-up (FU) observations were divided into 3 groups (short-term FU = 0–3 months; intermediate FU = 3–12 months; and long-term FU = more than 12 months). Table 1 and Table 2 summarize the baseline characteristics of the included study participants. Due to the limited availability of the data, all types of articles were primarily considered, including case series, to integrate a patient population and an amount of information as large as possible. Most published studies available are mainly uncontrolled prospective or retrospective studies, case series, and case reports, limiting high-quality evidence. Unfortunately, no randomized controlled trials (RCTs) have yet been conducted on this topic. Two authors were contacted via email in the context of data collection and asked to share more detailed measurement data with us as the scope or accuracy of the data presented in the version of the publication available to us was not sufficient for data analysis [16,17,18]. Finally, we included 18 publications, with a total number of 70 patients, in our study. The following PRISMA flow diagram provides an overview of the selection process (Figure 1).

### 2.2. Statistical Analysis

According to the 3 pre-defined follow-up periods, we determined VAS and TUG values, given as the mean and standard deviation. Because a traditional meta-analysis may be inappropriate due to confounding factors, we calculated a Cohen’s D effect size for each of the 3 follow-up groups to assess the variables relative to each study’s own control. As the sample size of the articles was often one patient, the standard deviation could not be estimated, and thus, Cohen’s D is undefined for single articles. Nevertheless, we added a table containing the overall Cohen’s D. Cohen’s D was calculated for all the patients from all articles.

## 3. Results

### 3.1. Study Cohort Characteristics and Data Presentation

The 70 SCS-PD patients included in this study were characterized by an inter-individual variability as 45 (64 %) PD patients suffered from co-incidental chronic pain disorder compared to 25 (36 %) PD patients without a history of chronic pain who received SCS due to their motor impairments. Chronic pain disorders cover the following diagnoses: neuropathic pain (NP), failed back surgery syndrome (FBSS), lower limb pain (LLP), musculoskeletal pain (MSP), central pain (CP), lower back pain (LBP), leg pain (LP), abdominal pain (AP), sensitivity disorders (SDOs), lumbar Spinal Stenosis (SSL), painful camptocormia (PC), painful Pisa Syndrome (PPS), and vascular pain (VP). The PD-related gait impairment was classified as motor impairment (MI), postural instability and gait disturbance (PIGD), and freezing of gait (FOG).

The study cohort distribution was the following: 31 males (pain: 13; no pain: 18), 22 females (pain: 17; no pain: 5), and 17 patients (pain: 15; no pain: 2) without a specified gender. Patients’ ages covered a range of 43–85 years (pain: 43–81 years versus no pain: 53–85 years), and disease duration or time since diagnosis ranged from 2–31 years (pain: 3–31 years versus no pain: 2–31 years) (Table 1 and Table 2).

### 3.2. Spinal Cord Stimulation Parameters and Implantation Protocol

The targeted spine level showed a wide variation, ranging from a high cervical spine level (C2) to low thoracic levels (Th10/11). The spine level of intervention can be divided into two groups (33% cervical/high thoracic SCS versus 66% lower thoracic SCS). The vast majority applied tonic SCS waveforms operating between 100 and 130 Hz (T-SCS), while burst SCS (B-SCS; 40 Hz) was used in three studies only. Notably, although there were two case reports in which cycle the SCS mode (SCS on/off in a pre-defined time pattern) was applied, SCS was used chronically (24 h) as these physical SCS properties are of relevance for the dose–response relationship and may have a considerable impact on the reported SCS outcome.

Applied SCS waveforms ranged from 5–130 Hz, 0,45–5.8 V/0.3–1.1 mA, and 60–500 µs for the PD patients with pain versus 15–300 Hz, 0.45–4.6 V, and 90–400 µs for the PD patients without pain. The two authors executing cycle SCS modes used different off-and-on periods. While Chakravarthy et al. compared T-SCS, B-SCS, and the cycle mode to 15–30 s SCS Off and 10–15 s SCS On with the standard B-SCS setting described above, Lai et al. compared 30 min SCS On versus 15 min SCS Off with a 60 Hz setting as a second SCS array implantation was required due to the dislocation of the first SCS implant [17,18].

DBS, as a previously performed cranial neuromodulation therapy, was present in 26 PD patients (with pain: 18 versus without pain: 8). It was also used for SCS or switched off during assessment as a comparison (Table 1 and Table 2).

### 3.3. Clinical SCS Outcome Divided Into Short-Term, Intermediate, and Long-Term Follow-Up

Apparently, the observational period of the described SCS effects on gait and pain in PD patients differs considerably, representing a considerable biasing confounder. Thus, the authors presented the data as three observation periods (short-term FU = 0–3 months; intermediate FU = 3–12 months; and long-term FU = more than 12 months) (Table 3 and Table 4).

#### 3.3.1. Short-Term SCS Effects (0–3 Months of FU)

Nearly all included participants perceived a meaningful clinical improvement related to gait and pain levels. In particular, in the VAS, FOG, and timed walking tests, these SCS effects could be detected, albeit to varying degrees (Table 3 and Table 4). However, Thevathasan et al. failed to demonstrate an improvement in gait parameters after SCS implantation [21]. Later studies, however, yielded more positive results. For example, Zhou et al. were able to record an improvement across different gait tasks (TUG 7 m, FOG, and MDS-UPPDRS III) 3 months after SCS therapy. Notably, another study found that a combination of SCS and medication was superior to SCS at baseline and after SCS was quantified by the MDS-UPDRS III [26,29]. These positive results (UPDRS/H&Y improvement) are in line with further uncontrolled in-human studies in the field. In contrast, Hubsch et al. showed that SCS alone—without medication—improved MDS-UPDRS III as well as the time/stride length in the SWS test, which further improved with adjunctive medication. However, no significant differences were measured in the FOG-Q when comparing SCS alone versus SCS with medication [24,32]. Lai et al. confirmed early-onset SCS effects on both the symptom complex and observed improvements across VAS, UPDRS III, and the TUG test. Hardware-associated failure in terms of dislocation became apparent by increased pain levels and a deteriorated gait, but they immediately improved after SCS revision [20].

#### 3.3.2. Intermediate SCS Effects (3–12 Months of FU)

Overall, we extracted data for 15 patients suffering from various types of pain (Table 3 and Table 4). For instance, Agari et al. confirmed an improvement in VAS, UPDRS III, TUG, and in the 10 m walk test at his second follow-up lasting up to 12 months. Clinical motor improvement was reduced by approximately half in all the scores 3 months after SCS implantation compared to baseline, apart from sustained pain control [28]. Similar results with a considerably lower decrease in function were shown by Nishioka and Nakajima, who were still able to demonstrate an improvement in UPDRS III and VAS in their three patients after 12 months as well. However, the pain-reducing effect (VAS) was somewhat less effective in two patients compared to the first follow-up after 2 weeks. In contrast, the therapeutic benefit for pain management was even greater in the third patient after 12 months. The H&Y scores remained largely constant in all three patients within the study period [31].

Using low thoracal B-SCS (Th 10 - 12) as a treatment for PD patients suffering from lower back pain, Furusawa and colleagues noted a significant reduction in FOG in two patients at the 24-week follow-up. In addition, persistently faster times in the TUG test compared to the baseline values over four to 24 weeks (23.1 ± 11.9 s, 21.3 ± 10.9 s, and 12.7 ± 4.7 s) were observed, similar to VAS and UPDRS [33].

Pinto de Souza et al. used SCS treatment on four patients with PIGD symptoms who were previously treated with thalamic DBS and found an improvement in different gait tasks after 6 months with adjunctive DBS therapy without medication, while additional medication led to increased motor performance displayed by the UPDRS-III (19.7 ± 67 versus 15.0 ± 4.2), along with a reduction in levodopa equivalent daily dose (LEED) from 812.5 mg/d ± 469.7 mg/d to 762.5 mg/d ± 423.0 mg/d [22].

#### 3.3.3. Long-Term SCS Effects (>12 Months of FU)

Chakravarthy et al. assessed 15 PD patients suffering from neuropathic pain (NP) and inserted thoracal leads in 14 patients versus a cervical lead in 1 patient using different SCS patterns (T-SCS versus B-SCS/chronic versus cycle SCS modes) and found no difference before and after SCS therapy (UPDRS/H&Y) (Table 3 and Table 4). However, after 22 months (4–33 m), −48 % to −67 % reduction in VAS was achieved depending on the subgroups; in contrast, 12% gait amelioration was present in 8/12 patients in the 10 m walk test and in 7/11 PD patients assessed by the TUG. With regard to the SCS pattern used, 21% received T-SCS, while 18% were stimulated with B-SCS. Interestingly, the cycle mode (alternating SCS off/on) showed a worsening effect in the 10 m walk test [19]. Like Chakravarthy, Landi et al. did not detect any improvement in the UPDRS III, contrary to another group. In this case, the UPDRS III changes were sustained up to the 24-month follow-up compared to the 12-month follow-up and baseline [29,30].

These prolonged SCS effects were observed in additional in-human trials. In their 3-year follow-up, in which four patients were included, Samotus et al. observed an improvement in UPDRS-III (32 ± 12 versus 26.2 ± 9.6) and the FOG-Q (20.0 ± 1.0 versus 16.8 ± 2.9). These effects remained stable even under L-dopa dose reduction [23,25,35].

### 3.4. MSA-P Patients

Some studies included participants other than those diagnosed with classic PD, classified as atypical Parkinson’s Disease variant and/or multisystem atrophy-associated Parkinson’s Disease (MSA-P). In a small-scale trial, the TUG test was significantly reduced in five MSA-P patients, while the UPDRS remained unchanged [23]. Zhang et al. described an additional case of one MSA-P patient with leg pain, in whom SCS had a positive effect on pain perception, FOG, and the UPDRS III [34].

## 4. Discussion

The therapeutical impact of spinal cord stimulation in chronic pain has been used for decades as an adjunctive interventional treatment option. However, the neurotherapeutic value of SCS applied to target gait impairment in PD patients remains unknown, displayed by the heterogenous SCS responsiveness published.

### 4.1. SCS as an Adjunctive Therapy for PD

After Melzack and Wall laid the foundation for the new therapeutic approach of SCS with their gate control theory, a neuromodulatory effect of the spinal cord stimulation on pain perception, transmission, processing, and dorsal column stimulation was reported for the first time in 1967 [36,37]. Since then, SCS has confirmed its raison d’être because of its potential as an additional therapy for chronic pain patients, yielding sustained pain relief in interventional pain management. In a preclinical study, Fuentes et al. were able to identify a positive effect on locomotion in Parkinson’s Disease in a rat model in 2009. Later, an improvement in motor deficits was also observed in the primate model [38,39,40]. However, the first-in-human approach failed to relieve akinesia and was not able to restore locomotion [21].

Backed up by these initial experimental observations, SCS has been studied to improve motor performance in PD patients due to its potentially positive effect on motor impairments described in the preclinical trials above, along with the pain-relieving effect. Although pain is not one of the typical cardinal motor symptoms of the disease, it represents a major burden for PD patients. The percentage of patients suffering from chronic pain disorders among PD patients ranges from 40% up to 83% [28,41,42].

This includes different types of pain, displayed in Table 1. While some symptoms may be directly associated with Parkinson’s Disease, such as painful motor fluctuations due to the wearing-off phenomenon or PD-associated chronic poor posture like Captocormia, pain in the form of musculoskeletal and neuronal pain, or FBSS, occurs independently of Parkinson’s Disease [27,28,41]. Fénelon et al. published the first positive case report describing the effects of SCS on motor impairment in a PD patient [27]. However, the quality of evidence supporting the use of SCS in PD patients is still low.

### 4.2. Mechanism of Action of SCS in PD-Associated Gait Impairment

#### 4.2.1. Pathophysiology of PD and Gait Disturbance

**Figure 2:**
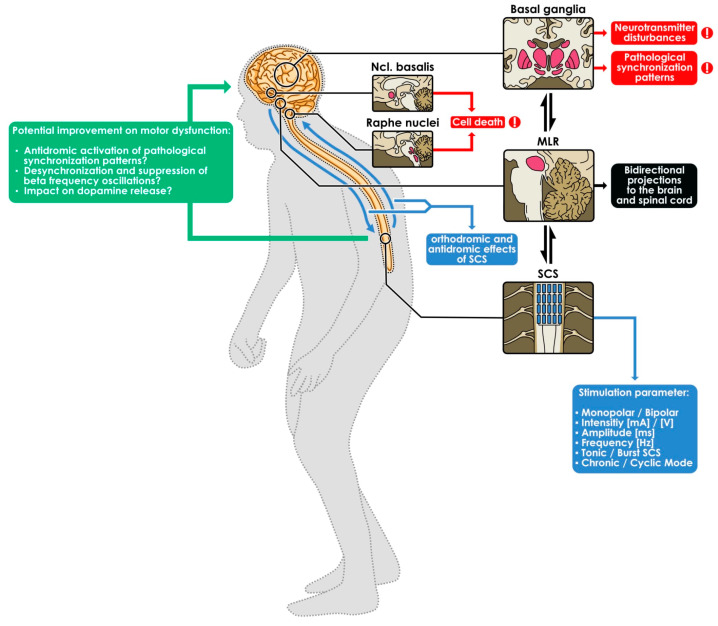
Schematic illustration of the effect of spinal cord stimulation on gait disturbance.

It has been assumed that movement impairment in PD patients occurs as a consequence of dysfunctional dopaminergic direct and indirect motor inhibitory control loops involving the substantia nigra pars compacta, striatum, globus pallidus internus, substantia nigra pars reticularis, and thalamus, relevant to the inhibition of the GPi and the substantia nigra pars reticularis (Figure 2). A dopamine deficiency resulting from other diseases or as a side effect of anti-dopamine medication could result in similar symptoms and lead to atypical parkinsonism or parkinsonoids, such as in the context of MSA-P [43].

The loss of dopaminergic inhibition of the motor inhibitory areas of the striatum and the GPi/GPe leads to disinhibition, resulting in pathologically increased motor inhibition and bradykinesia. However, the dopamine system is not the only system affected by this disorder. The resulting functional excess of acetylcholine-producing neurons within the loop additionally intensifies the motor symptoms, resulting in tremors.

In addition, several other regulatory imbalances with different onset can occur, involving the acetylcholinergic, serotonergic, and noradrenergic systems, along with cell deaths, such as in the raphe nuclei or the nucleus basalis, causing the pleiomorphic symptoms associated with Parkinson’s Disease [44]. The decrease in neurons and activity has been demonstrated in neuroimaging studies, e.g., positron emission tomography (PET) [45].

In the classic Parkinson’s model, it was primarily assumed that a pathologically increased output in the basal ganglia loop associated with PD was the cause of the resulting dysfunctional movement [43]. Currently, it is more common to attribute a greater role to other parameters, such as pattern and synchronicity, in addition to the firing rate. However, PD-associated changes are modifications in the neuronal activity of individual cells and result in various synchronization effects [46,47,48,49,50,51].

#### 4.2.2. Mechanism of Action of SCS for PD-Associated Gait Impairment

In general, several theories exist for possible pathways through which SCS may interact with spinal and supraspinal circuits relevant to PD gait physiology and gait impairment. However, the precise pathways remain largely unknown and are currently a matter of ongoing preclinical and clinical discussions [50,52,53,54]. SCS may evoke an antidromic activation of the pathological synchronization patterns, described above, involving the direct and indirect motor inhibitory control loops of the substantia nigra pars compacta, striatum, globus pallidus internus, substantia nigra pars reticularis, and thalamus, relevant to the inhibition of the GPi and the substantia nigra pars reticularis. Other brain circuits relevant to gait and posture include but may not be limited to the mesencephalic area, especially the mesencephalic locomotor region (MLR), including the cuneiform nuclei and PPN. In addition to the antidromic activation already mentioned, SCS may also have a positive influence on motor impairment via the activation of afferent sensory pathways of the dorsal column of the spinal cord. These signaling pathways appear to be of relevance for the pathophysiological important central areas of motor control (Figure 2). One possible pathway involves the desynchronization and suppression of beta frequency oscillations, thus permitting movement initiation and the physiological execution of conscious movement patterns. Notably, DBS and SCS have been shown to impact dopamine release [50,55,56]. Given these preliminary experimental findings, it seems that additional brain circuits involved in PD may be affected by spinal modulation via various signaling pathways (Figure 2).

Other invasive and noninvasive neuromodulation therapies have been additionally studied and include invasive deep brain stimulation (DBS) and non-invasive procedures like transcranial magnetic stimulation (TMS), trans-spinal theta burst magnetic stimulation (TsMS), or cervical vagus nerve stimulation (VNS).

DBS targeting different brain circuits and applying different stimulation patterns (e.g., low-frequency STN-DBS) has been investigated to restore PD gait and postural instability. For instance, the pedunculopontine nucleus has gained increased clinical attention as the PPN bidirectionally projects to the spinal cord (antidromic/orthodromic effects) and upper order brain nodi (e.g., GPe, GPi, and SNr) (Figure 2) However, inconsistent and conflicting findings call for the conceptualization of additional rigorous study protocols to determine its therapeutic usefulness [13,57].

Motor symptoms of Parkinson’s Disease are caused by alterations of neurotransmitter balance in the basal ganglia (e.g., changes in the dopaminergic and acetylcholinergic signaling pathways), which may lead to pathological synchronization patterns, among other things. In addition to neuron loss in the nucleus basalis and the raphe nuclei, the mesencephalic locomotor region (MLR) and the cuneiform and pedunculopontine nucleus (PPN) and its reciprocal cerebral and spinal connections are negatively affected (right side of Figure 2. Spinal cord stimulation (SCS) could exert a positive influence on gait disturbance via orthodromic effects on afferent pathways of the dorsal column medial lemniscus system, with resulting antidromic effects on cerebral and extrapyramidal efferents (blue arrows in the left side of Figure 2).

Noninvasive spinal modulation has been most recently used, particularly TsMS in a randomized double-blind controlled trial. Menezes et al. applied Ts MS on the spine level Th3 operating at 100% of the motor threshold in 33 PD patients suffering from gait disorders and postural instability and assessed different gait tasks (total TUG, UPDRS III, dual-task TUG, FOG, and PDQ-39). In summary, verum TsMS was found to not be superior to sham TsMS, along with a low rate of temporary adverse events (insomnia and urinary incontinence). These observations were in line with results derived from a meta-analysis [58,59]. An improvement was recorded for FOG and UPDRS (especially, gait, other axial scores, and bradykinesia), whereas no significant differences have been reported for TUG and Turning Time and Steps [13].

In additional in-human studies, cervical non-invasive VNS was found to be well tolerated (safety profile) and to improve sub-tasks of gait in PD patients in a pilot uncontrolled open-label study. These preliminary results were confirmed under a rigorous study protocol comparing varum versus sham VNS, thus indicating that VNS may have an effect on neuroplasticity and antidromic excitation conduction with the activation of brain circuits relevant to gait initiation [13,60].

### 4.3. Limitations and Future Directions to Improve SCS Outcome in PD-Associated Gait Impairment

The data presented in this systematic review disclose several confounding variables, limiting a conclusion in favor or against adjunctive SCS therapy for the treatment of PD-associated gait and postural instability. First, the published studies differ regarding implantation techniques (open surgical paddle-type lead versus percutaneous implanted cylindric leads, trial stimulation, and duration of trial stimulation), the targeted spine level (from lower cervical to lower thoracic spine), stimulation parameters relevant to the dose–response relationship (tonic 100–130 Hz, burst 40 Hz, and chronic mode versus cycle mode), different outcome measures, the short-term follow-up period, and the co-incidence of chronic pain. Although we assessed a large cohort of PD patients in our review, the vast majority of the findings were derived from uncontrolled observational studies, case series, and case reports and may thus be prone to give positive results and outcomes and be characterized by poor-quality evidence [61,62,63,64,65]. The authors underwent efforts to counterbalance this data search and selection bias and addressed this by directly approaching the corresponding authors to request relevant clinical data that were missing.

Activity-dependent spinal cord stimulation targeting the dorsal root entry zone, combined with extensive neurorehabilitation, prompted encouraging results, demonstrating its capability to restore gait function in individuals with spinal cord injury (paraplegic). This innovative and translational closed-loop SCS approach permits highly selective spatial-temporal neurostimulation during intended leg movements. In a most recent case report, Milekovic et al. were able to demonstrate that activity-dependent SCS improved gait in a PD patient, thus having the potential to address open questions and gaps regarding the use of SCS for PD-associated locomotor deficits [66,67]. These nascent developments and innovations in the field of neural engineering and computational neuroscience have the potential to overcome current barriers and counteract limitations in interventional neurostimulation therapies and represent an important step in future research [68,69].

## 5. Conclusions

Conclusively, the available literature does not support the clinical use of SCS to restore gait and postural instability in PD patients but confirms its usefulness as an interventional pain management therapy. However, this may not limit the valence of its potential therapeutic value as new innovative SCS technologies are on the horizon. Hence, the authors advocate for the re-assessment of SCS in a large-scale and rigorously designed study protocol. Notably, while SCS for pain is approved in the US and Europe, SCS for PD gait disorders alone is not and is thus being used “off-label”.

Although not within the scope of this systematic review, it is worth mentioning that non-invasive neurotherapeutics targeting central or spinal structures (e.g., TsMS and nVNS) were found to ameliorate gait performance in PD patients. Like invasive SCS, randomized controlled studies are warranted to evidently demonstrate the efficacy and safety of TsMS and nVNS.

## Figures and Tables

**Table 1:** Spinal cord stimulation in PD patients without a history of chronic pain.

Study Name (No. of Included Patients)	Gender (m/f/d); Age	Disease Duration (Age at Diagnosis)	Spine Level; Stimulation Parameters +/− DBS	Indication	Medication (Pre- vs. Post- Medication)	Outcome Measures	Baseline	1st Follow-Up	Last Follow-Up
**[21]** **(2 p)**	75 y	/	**high cervical** (**A**) 130 Hz/2 V/240 µs (**B**) 130 Hz/3 V/240 µs	MI **n.s.**	**n.s.**	**UPDRS III** **10 m walk** (s)	**means:** **A/B** 0.68 37.8 ± 11.5 5.5 ± 1.2	**10 d** **A** 1.9/**B** 2.1 **A** 35.4 ± 12.5 **B** 37.3 ± 10.5 **A** 5.4 ± 0.4 **B** 5.6 ± 1.0	/
	77 y	/	**high cervical** (**A**) 300 Hz/3 V/200 µs (**B**) 300 Hz/4 V/200 µs	MI **n.s.**	**n.s.**	**UPDRS** **10 m walk** (s)	means: **A/B** 0.68 37.8 ± 11.5 unable	**10 d** **A** 1.9/**B** 2.1 **A** 35.4 ± 12.5 **B** 37.3 ± 10.5 unable	/
**[22]** **(4 p)**	3/m (64, 68, 69 y) 1/f (56 y)	21.2 y (± 10.1)	**T2–T4** 300 Hz, 2–4.6 V, 90 μs **+ DBS** (4 p)	PIGD **n.s.**	LEED (mg/d) 812.5 ± 469.7 **vs.** 762.5 ± 423.0	**UPDRS III** **TUG** (s) **20 m walk** (s) **FOG-Q**	(DBS on/ Med off) 33.0 ± 13.7 35.2 ± 23.4 94.0 ± 72.0 17.7 ± 0.9	**6 m** (DBS on/ Med off) 19.7 ± 67 11.8 ± 5.9 29.0 ± 14.0 7.7 ± 0.9	**6 m** (DBS on/Med on) 15.0 ± 4.2
**[23]** **(5 p/4 p)**	5/m 71 y (63–85 y)	14 y (8–18 y)	**T8–10** 30–130 Hz, 300–400 μs	FOG	L-Dopa(mg/d) 1330 ± 441 **vs.** **6 m** 1215 ± 420 **vs**. **36 m** 1262.5 ± 619.3	**UPDRS III** **Sit-to-stand** (s) **FOG-Q**	32 ± 12 7.6 ± 6.0 20.0 ± 1.0	**6 m (5 p)** 21.4 ± 10.8 3.8 ± 2.5 15.0 ± 3.9	**36 m (4 p)** 26.2 ± 9.6 n.s. 16.8 ± 2.9
**[24]** **(5 p)**	5/m 68.8 ± 3.9 y	14.8 ± 7 y	**T10–11** **T-SCS** 100 Hz and 300 μs **+ DBS** (1 p)	FOG	**n.s.**	**MDS-UPDRS** **III** **SWS** Time (s) Steps **FOG-Q**	No significance	**60 d** Med off/ stim on −23.22% Med off/ stim on −23.6% −12.4% Med on/stim off −19.3% −18%	Med on/stim on −36.8% Med on/stim on −29.8% −20%
**[25]** **(8 p)** **PD Group** **(3 p)**	3/m 64 ± 11 y	8 ± 5 y	**C2–5** 20–40 Hz/1–2.5 V/120–280 µs **+ DBS** (1p)	PIGD	**LEED** 550–1164.25 mg/d	**MDS-UPDRS III** **TUG: 5 m** Time (s) Step length (cm)	44 ± 5 **Med off**	**3 m** 39 ± 3 −15% unchanged	**6 m** 37 ± 1 −14% +20%
**MSA-P Group** **(5 p)**	2/m 3/f 60 ± 5 y	4 ± 2 y	**C2–5** 15–45 Hz/0.45–3.8 V/120–310 µs **+ DBS** (2 p)	PIGD	**LEED** 0–600 mg/d	**MDS-UPDRS III** **TUG: 5 m** Time (s) Step length (cm)	44 ± 16 **Med off**	**3 m** 44 ± 14 −40% −8%	**6 m** 44 ± 18 −28% −10%
**[26]** **(1 p)**	F 66 y	15 y 51 y	**T9–10** 260 Hz, 1.0 V, 210 µs	FOG	**n.s.**	**MDS-UPDRS** **III** **TUG: 7m** Time (s) Steps **FOG** (NFOGQ)	Med off Med on 33/19 31 63 22	**3 m** Med off/Med on 12/8 **1 d** 9 14 **3 m** 6	

A/B-A—subthreshold SCS; B—suprathreshold SCS; AP—abdominal pain; B-SCS—burst spinal cord stimulation; CP—central pain; FBSS—failed back surgery syndrome; FOG—freezing of gait; FOG-Q—Freezing of Gait Questionnaire; HY = Hoehn Yahr Score; Hz—Hertz; LBP—lower back pain; LEED—levodopa equivalent daily dose; LLP—lower limb pain; LP—leg pain; m—month; MDS-UPDRS III—Movement Disorder Society United Parkinson Disease Rating Scale, motor score; Med—medication; MI—motor impairment; min—minutes; ms—milliseconds; MSA-P—multiple system atrophy with predominant parkinsonism; MSP—musculoskeletal pain; μs—microseconds; NP—neuropathic pain; n.s.—not specified; p—patient(s); PC—painful camptocormia; PD—Parkinson’s Disease; PIGD—postural instability and gait disturbance; PPS—painful Pisa Syndrome; SD—standard deviation; SDO—sensitivity disorder; Stim—stimulation; SSL—lumbar Spinal Stenosis; SWS—stand-walk-sit test; T-SCS—tonic spinal cord stimulation; TUG—Timed Up and Go test; UPDRS—United Parkinson Disease Rating Scale; UPDRS III—United Parkinson Disease Rating Scale, motor score; V—volt; VAS—Visual Analog Scale; VP—vascular pain; vs. = versus; and y—years.

**Table 2:** Spinal cord stimulation in PD patients with a history of chronic pain.

(No. of Included Patients)	Gender (m/f/d); Age	Disease Duration (Age at Diagnosis)	Spine Level; Stimulation Parameters +/− DBS	Indication	Medication (Pre- vs. Post-Medication)	Outcome Measures	Baseline	1st Follow-Up	Last Follow-Up
**[27]** **(1 p)**	M 74 y	5 y 69 y	**T9–T10** 100–130 Hz/ 3.5 V/410 µs	NP (LLP) FBSS	L-Dopa (1200 mg/d) **vs.** **n.s.**	**VAS** **UPDRS III** **7 m walk** (s)	Med off/Stim off 6.9 ± 1.0 56.7 ± 3.3 29.3 ± 2.3	**30 min–60 min** Med off/ Stim on 1.9 ± 0.2 29.7 ± 2.5 23 ± 6.3	**30 min–60 min** Med on/ Stim off 4.5 26 22
**[28]** **(15 p)**	5/m 10/f 63–79 y (mean 71.1 y)	7–31 y (mean 17.2 y)	**T7–T12** 5–20 Hz/0–4 V/210–330 µs **+ DBS** (7 p)	MSP NP CP LBP LP AP	**n.s.**	**VAS** **UPDRS III** **TUG** (s) **10 m walk** (s)	8.9 (7.8–10) 23.5 ± 9.7 21.6 ± 10.7 14.7 ± 8.4	**3 m** 2.0 ( 0–3.3) 18.9 ± 10.4 15.6 ± 7.3 12.7 ± 8.0	**12 m** 2.3 ( 0–4) 21.3 ± 12.2 18.2 ± 10.8 13.3 ± 9.3
**[29]** **(1 p)**	F 65 y	**n.s.**	**T9–10** 60 Hz, 1.5 V, 100 μs **vs.** 30 Hz; 1.8–2.5 V, 250-μs **+ DBS**	LP NP SDO	L-Dopa (750 mg/day) **vs.** **n.s.**	**VAS** **UPDRS III** **20 m walk (s)**	9–10 Med off/stim off 77 Med on/stim on 23 Med on/stim off 57	**1 d** −50% unchanged unchanged unchanged	**16 m** −70% unchanged unchanged unchanged −20%
**[30]** **(1 p)**	F 43 y	8 y 35 y	**C2** 40 Hz, 0.3 - 1.1 mA, 500 μs	NP	carbidopa L-Dopa rasagiline **n.s.**	**VAS** **UPDRS III** **10 m walk (s)**	8–9 28 17	**1 w** 2 **12 m** 22 n.s.	**24 m** 0–2 16 11
**[31]** **(3 p)**	f 67 y	5 y 62 y	**T8–L1** 20–65 Hz, 0.6–2.0 V, 360–420 μs	FBSS SSL LBP LLP		**VAS** **UPDRS III** **HY**	10 35 4	**2 w** 7 **n.s.** 4	**12 m** 4 27 4
	m 80 y	10 y 70 y	**T8–L1** 5 Hz, 0.45–5.8 V, 60–450 μs	SSL LBP LLP		**VAS** **UPDRS III** **HY**	7 43 5	**2 w** 0 **n.s.** 4	**12 m** 4 29 4
	m 76 y	13 y 63 y	**T8–L1** 5–10 Hz, 0.6–1.7 V, 420 μs	SSL LBP LLP		**VAS** **UPDRS III** **HY**	9 33 4	**2 w** 2 **n.s.** 3	**12 m** 3 18 4
**[32]** **(1 p)**	f 65 y	12 y 53 y	**T8** **Program A:** 7 Hz, 2,5 V, 450 μs **Program B:** 7 Hz, 3,5 V, 250 μs **+ DBS**	PC PPS	800 mg/d carbidopa/L-Dopa, 4.5 mg/d pramipexole 20 mg/d istradefylline	**VAS** **UPDRS** **TUG** (s)	10 (p.o. 2) 48 15	**11 d** 2 33 8	**29 d** / 34 7
**[16]** **(1 p)**	m 74 y	3 y 71 y	**T6–8** 40 Hz burst, 500 Hz intra-burst rate, 1 ms	LBP LP	L-dopa/carbidopa, selegiline, ropinirole hydrochloride **n.s.**	**Pain** ( SF-MPQ-2) **UPDRS III** **20 m walk (s)** **HY**	47 20 32 3	**Post** **stimulation** 18 **2 w** 6 25 2	
**[19]** **(15 p)**	/ 74 y ±5.2	17 y ±8.7	**14 Thoracic** **1 cervical** - **T-SCS** (40 Hz 350 μs; 10 Hz 350 μs) - **B-SCS** (40 Hz, 500 Hz, 1 ms) five-pulse train - **Cycle mode** (on-time of 10– 15 s; 40 Hz, 500 Hz intra-burst rate, 1 ms) **+ DBS** (8 p)	NP	**n.s.**	**VAS** **10 m walk** (s) ( 11 p) **TUG** (s) (11 p) **UPDRS + HY**	No difference	**22 m** **( 4–33 m)** prior **DBS**/ no DBS −61%/−57% 8/11 p 12% improvement 7/11 p 21% improvement	**Cycling/burst** −67%/−48% **Continuous burst:** 18% improvement **Cycling**: 7% worsening
**[33]** **(5 p)**	2/m 3/f 74 y 66–81 y	12.4 y 5–31 y	**T8–9** **B-SCS** 40 Hz burst, 500 Hz intra-burst rate, 1 ms	LBP	**n.s.**	**VAS** **UPDRS III** **TUG** (s) **FOG** (3 p: P1, P2, P3)	64.6 ± 30.3 34.6 ± 12.8 23.1 ± 11.9 3/2/1	**4 w** 32.4 ± 28.3 23.8 ± 4.4 21.3 ± 10.9	**24 w** 57.0 ± 33.2 25.0 ± 9.7 12.7 ± 4.7 1/0/0
**[20]** **(1 p)**	M 73 y	13 60 y	**1st** **T8–10** **continuous** 30 Hz, 180–210µs, 1.5–2.5 V **Dislocation** **2nd** **T8–9** **cycling** 30 min on and 15 min off 60 Hz, 270–390µs, 3.6–4.0 V + **DBS**	FBSS LBP	**n.s.**	**VAS** **UPDRS III** **TUG** (s)	(Med OFF/Med ON) 8/7 45/43 40/22	**1st SCS** **2 d** 3/3 **2 m** 7/6 **2 d** 43/43 **2 m** 45/45 **2 d** 26/23 **2 m** 29/22	**2nd SCS** **4 d** 3/3 **2 m** 3/3 **4 d** 42/42 **2 m** 42/40 **4 d** 26/22 **2 m** 22/22
**[34]** **(1 p)** **(MSA-P)**	M 75 y	5 y 70 y	**T10–12** 60 Hz/1.0 V/200 µs	LP	**n.s.**	**VAS** **MDS-UPDRS III** **FOG** (NFOGQ)		**6 m** −71% −38% −63%	

A/B-A—subthreshold; B—suprathreshold; AP—abdominal pain; B-SCS—burst spinal cord stimulation; CP—central pain; FBSS—failed back surgery syndrome; FOG—freezing of gait; FOG-Q—Freezing of Gait Questionnaire; HY = Hoehn Yahr Score; Hz—Hertz; LBP—lower back pain; LEED—levodopa equivalent daily dose; LLP—lower limb pain; LP—leg pain; m—month; MDS-UPDRS III—Movement Disorder Society United Parkinson Disease Rating Scale, motor score; Med—medication; MI—motor impairment; min—minutes; ms—milliseconds; MSA-P—multiple system atrophy with predominant parkinsonism; MSP—musculoskeletal pain; μs—microseconds; NP—neuropathic pain; n.s.—not specified; p—patient(s); PC—painful camptocormia; PD—Parkinson’s Disease; PIGD—postural instability and gait disturbance; p.o.—postoperative; PPS—painful Pisa Syndrome; SD—standard deviation; SDO—sensitivity disorder; SF-MPQ-2—Short-Form McGill Questionnaire 2; Stim—stimulation; SSL—lumbar Spinal Stenosis; SWS—stand-walk-sit test; T-SCS—tonic spinal cord stimulation; TUG—Timed Up and Go test; UPDRS—United Parkinson Disease Rating Scale; UPDRS III—United Parkinson Disease Rating Scale, motor score; V—volt; VAS—Visual Analog Scale; VP—vascular pain; vs. = versus; and y—years.

**Table 3:** VAS (mean and standard deviation) and overall Cohen’s D of VAS and its 95% confidence interval at different defined follow-up periods (T1 = 0–3 months; T2 = 3–12 months; and T3 = more than 12 months) of included studies reporting pain outcome score measures.

VAS	Base line			T1			T2			T3			T4		
References	n	Mean	Sd	n	Mean	Sd	n	Mean	Sd	n	Mean	Sd	n	Mean	Sd
[21]	1	0.7		0			0			0			0		
[28]	1	8.9		1	2		1	2.3		0			0		
[30]	1	8.5		1	2		1	0.0		1	0.0		0		
[34] [31]	3	8.7	1.5	3	3	3.6	3	3.0	3.6	3	3.7	0.6	0		
[32]	1	10.0		1	2		0			0			0		
[20]	1	7.0		1	6		1	3.0		0			0		
VAS overall effect size				n	Cohen’s D				95% CI						
T1				6	1.96				[0.51–3.34]						
T2				5	2.56				[0.77–4.27]						
T3				4	2.61				[0.56–4.56]						
T4				0											

**Table 4:** TUG values (mean and standard deviation) and overall Cohen’s D of VAS and its 95% confidence interval at different defined follow-up periods (T1 = 0–3 months; T2 = 3–12 months; and T3 = more than 12 months) of included studies reporting pain outcome score measures.

TUG	Baseline			T1			T2			T3			T4		
References	n	Mean	Sd	n	Mean	Sd	n	Mean	Sd	n	Mean	Sd	n	Mean	Sd
[30]	0			1	28.0		1	22.0		1	16.0		0		
[31]	3	37.0	5.3	0			0			3	24.7	5.9	0		
[22]	4	33.0	13.7	0			0			4	19.8	6.8	0		
[32]	1	48.0		1	34.0		0			0			0		
[23]	5	32.2	11.7	5	21.4	10.8	0			0			0		
[20]	1	40.0		1	22.0		1	22.0		0			0		
[26]	1	31.0		1	9.0		0			0			0		
TUG overall effect size				n	Cohen’s D					95% CI					
T1				8	2.10					[0.83–3.33]					
T2				1											
T3				7	2.33					[0.90–3.7]					
T4				0											

## Data Availability

The original data presented in this study are openly available in the open science framework [OSF] at [https://doi.org/10.17605/OSF.IO/BRXYA].
